# Mechanisms promoting biodiversity in ecosystems

**DOI:** 10.1002/qub2.77

**Published:** 2024-10-26

**Authors:** Ju Kang, Yiyuan Niu, Xin Wang

**Affiliations:** ^1^ School of Physics Sun Yat‐sen University Guangzhou China

**Keywords:** biodiversity, competitive exclusion principle, paradox of the plankton

## Abstract

Explaining biodiversity is the central focus in theoretical ecology. A significant obstacle arises from the competitive exclusion principle (CEP), which states that two species competing for the same type of resources cannot coexist at constant population densities, or more generally, the number of consumer species cannot exceed that of resource species at steady states. The conflict between CEP and biodiversity is exemplified by the paradox of the plankton, where a few types of limiting resources support a plethora of plankton species. In this review, we introduce mechanisms proposed over the years for promoting biodiversity in ecosystems, with a special focus on those that alleviate the constraints imposed by the CEP, including mechanisms that challenge the CEP in well‐mixed systems at a steady state or those that circumvent its limitations through contextual differences.

## 1 INTRODUCTION

Biodiversity represents a fascinating aspect of life on Earth, encompassing both macroscopic and microscopic species inhabiting terrestrial and aquatic realms [1, 2, 3, 4]. In tropical forest ecosystems, thousands of plant and animal species coexist [3] with a gram of soil harboring 2000 or more microbial species [1]. In the sunlit zones of oceans, there are over 150,000 species of eukaryotic plankton [4]. However, explaining biodiversity is not a simple issue. In 2005, the question “What determined species diversity?” was listed in *Science* as one of 125 challenging scientific questions [2].

A significant obstacle in explaining biodiversity arises from the competitive exclusion principle (CEP), also referred to as Gause’s Law [5], which states that two species competing for a single resource cannot coexist at a steady state, as initially formulated by the theoretical foundation of Volterra [6] and the experimental studies of Gause [5]. In the 1960s, MacArthur and Levins extended the concept of CEP to ecosystems with an arbitrary number of resource species [7], stating that the number of consumer species cannot exceed that of the resources at steady states. In fact, the concept of CEP was further abstracted in mathematical formulations, replacing the context of resource species with limiting factors [8, 9]. However, for the clarity of discussion, definitions of CEP related to limiting factors are excluded in this review.

The conflict between CEP and biodiversity has been observed both in laboratories and in the wild. In nature, this conflict is exemplified by the famous paradox of the plankton, where in aquatic environments, very few types of abiotic resources support hundreds or more plankton species. In laboratories, Park reported in the 1950s that two different species of beetles coexisted for more than 2 years while competing for the same type of food (flour) [10]. Similarly, Ayala observed that two different species of flies cohabited for over 40 weeks while competing for the same abiotic resource in a bottle with serial transfer [11]. All these observations demonstrate violations of the CEP.

Over time, various mechanisms have been proposed to explain biodiversity in natural ecosystems. This review explores these mechanisms, focusing on those that alleviate the constraint of the CEP. Such mechanisms may either challenge the CEP in its original context, particularly in well‐mixed and steady‐state systems or bypass its limitations through contextual differences. For clarity, our review is structured as follows: we will first introduce the classical proof of the CEP raised by MacArthur and Levins [7], along with the scenarios and formulations that would undoubtedly fall under the constraint of the CEP, and then examine mechanisms that transcend the limits set by the CEP.

## 2 THE CLASSICAL PROOF OF CEP

**FIGURE 1:**
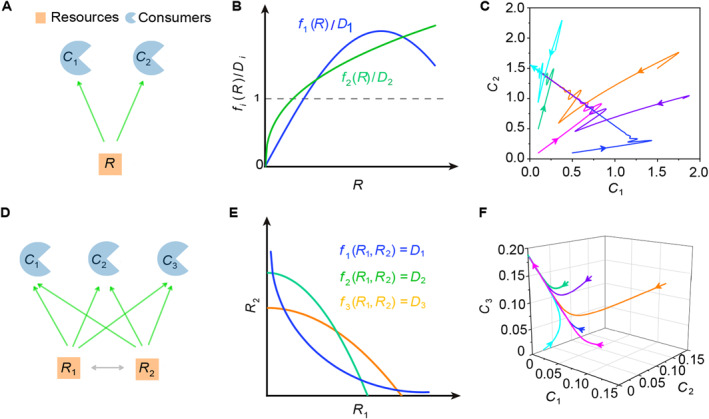
MacArthur and Levins’ classical mathematical proof of the CEP [7]. (A, D) Cases where *S*_*C*_ consumer species compete for *S*_*R*_ resource species. The green arrows denote biomass flow between consumption relationships, with predation or facilitation forbidden among consumers but allowed among resources (illustrated by gray arrows). (B, E) At a steady state, the coexistence of all consumer species demands the intersection of three lines at the same point, which is typically impossible. (C, F) Phase trajectories of consumer species that cannot coexist in a steady state. The arrows in (C, F) indicate the directions of time evolution. Figure redrawn from the study by Wang and Liu [12]. (C, E) were produced using the same equations as in the reference [12], but with slightly different parameters. CEP, competitive exclusion principle.

In the 1960s, MacArthur and Levin proposed a classical proof of the CEP for the generic case in which *S*_*C*_ consumer species compete for *S*_*R*_ resource species, wherein the CEP states that *S*_*C*_ ≤ *S*_*R*_ at steady state [7]. For convenience, first we rephrase the simplest case of two consumer species *C*_1_ and *C*_2_ competing for one resource species *R* (i.e., *S*_*C*_ = 2 and *S*_*R*_ = 1, see Figure 1A) in which they considered the population dynamics of the system via a consumer‐resource model in the following form:

(1) $$\left\{\begin{array}{l} {\dot{C}}_{i}=C_{i}\left(\mu_{i}\left(R\right)-D_{i}\right),i=1,2;\\ \dot{R}=g\left(R,C_{1},C_{2}\right). \end{array}\right.$$

here *μ*_*i*_ and *g* represent unspecified functions and *D*_*i*_ is the mortality rate of species *C*_*i*_. At a steady state, if both consumer species coexist, it is expected that *μ*_*i*_(*R*) = *D*_*i*_, where *i* = 1, 2. This demands that the two curves *y* = *μ*_1_(*R*)/*D*_1_ and *y* = *μ*_2_(*R*)/*D*_2_ intersect the line *y* = 1 at the same point, a situation that is typically unattainable (see Figure 1B), unless specific constraints on model parameters are met (with Lebesgue measure zero). Consequently, two consumer species generally cannot coexist with one type of resource at a steady state (see Figure 1C). In the case of *S*_*C*_ = 3 and *S*_*R*_ = 2, a similar proof strategy can be employed (see Figure 1D–F), and more broadly, this proof strategy can be applied to any of the cases with positive integer values of *S*_*C*_ and *S*_*R*_ [7].

## 3 SCENARIOS AND FORMULATIONS CONSTRAINED BY CEP AT STEADY STATE

### 3.1 Generalized Lotka–Volterra model is typically subject to the constraint of CEP

The pioneering work of Lotka [13] and Volterra [6] established the foundational mathematical models explaining the interactions of species, which were refined and expanded into the widely adopted Generalized Lotka–Volterra (GLV) model. The GLV model is a highly useful theoretical tool for modeling various types of relationships among different species. It is simple and intuitive, encompassing direct competition, trophic relations, direct influences on growth rates through mechanisms such as parasitism, and indirect interactions through secreted metabolites such as toxins. However, the GLV model omits interaction details within ecological communities, treating inhibitory interactions from mechanisms such as competition for food and inhibition by secreted toxins identically. Another influential modeling framework is the consumer–resource model proposed by MacArthur [14], which is mechanistic in modeling interaction details but introduces more variables for the explicit consideration of resource species.

Despite the extensive application of the GLV model [15, 16, 17, 18, 19, 20, 21], special attention needs to be paid to its application in tackling issues involving the CEP. This is due to the fact that freely chosen parameters in the GLV model implicitly imply subjection to the CEP [12]. The central idea could be illustrated using the simple case of the GLV model involving two types of competing species *C*_*i*_ (*i* = 1,2):

(2) $$\left\{\begin{array}{l} {\dot{C}}_{1}=C_{1}\left(\alpha_{1}-\beta_{11}C_{1}-\beta_{12}C_{2}\right),\\ {\dot{C}}_{2}=C_{2}\left(\alpha_{2}-\beta_{21}C_{1}-\beta_{22}C_{2}\right), \end{array}\right.$$

where *α*_*i*_ and *β*_*ij*_ (*i*, *j* = 1, 2) are parameters. Generally, in GLV models, there is no specific constraint on the choice of parameters *α*_*i*_, *β*_*ij*_. To illustrate the implicit subjection to the CEP, Wang and Liu [12] consider the consumer–resource model proposed by MacArthur [14] that can be compared to this case with two consumer species and one type of biotic resource (*S*_*C*_ = 2 and *S*_*R*_ = 1):

(3) $$\left\{\begin{array}{l} {\dot{C}}_{1}=\left(\alpha_{1}^{\prime }R-D_{1}\right)C_{1},\\ {\dot{C}}_{2}=\left(\alpha_{2}^{\prime }R-D_{2}\right)C_{2},\\ \dot{R}=\frac{r_{a}}{K_{a}}R\left[K_{a}\left(1-R/K_{a}\right)-\beta_{1}^{\prime }C_{1}-\beta_{2}^{\prime }C_{2}\right], \end{array}\right.$$

where $\alpha_{i}^{\prime }$, $\beta_{i}^{\prime }$, *D*_*i*_ (*i*, *j* = 1, 2), *r*_*a*_, and *K*_*a*_ are parameters. In fact, by assuming a fast equilibrium condition for resource species, Equation (3) can be reduced to Equation (2), with $\alpha_{i}=\alpha_{i}^{\prime }K_{a}-D_{i},\beta_{ij}=\alpha_{i}^{\prime }\beta_{j}^{\prime }$ (*i*, *j* = 1, 2). However, this leads to a pathological constraint on parameters $\beta_{ij},\;\text{given}\;\text{by}\;\frac{\beta_{11}}{\beta_{12}}=\frac{\beta_{21}}{\beta_{22}}$, severely restricting the choice of parameters in a GLV model to a zero‐measure parameter set. The above analysis applies to GLV models with multiple consumer species. A non‐zero parameter set for parameter choice exists only when these models are subject to the CEP, where the number of resource species exceeds the number of consumer species [12].

### 3.2 The scenario involving only chasing pairs is subject to the constraint of CEP

For well‐mixed ecosystems, the population dynamics of the system can be rigorously derived from microscopic species interactions using mean field theory borrowed from statistical physics. This method is widely used in studying chemical reactions and is suitable for ecological systems if the consumer species can move freely. By applying this method, Wang and Liu investigated whether the scenario involving only chasing pairs is subject to the constraint of CEP at steady state [12].

**FIGURE 2:**
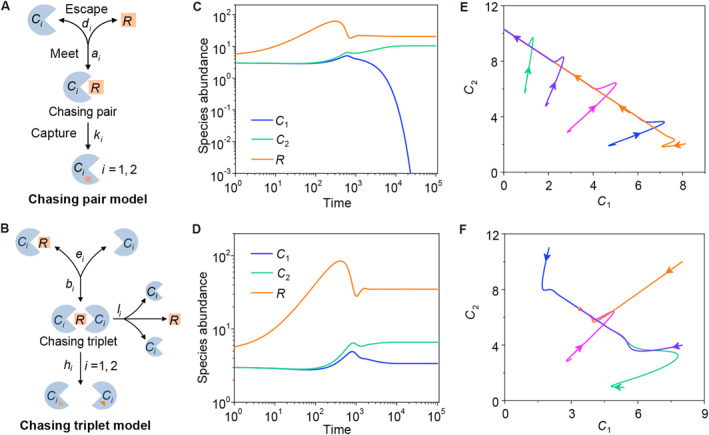
A scenario involving only chasing pairs is subject to the constraint of CEP, whereas further integration with chasing triplets can naturally break the CEP [12]. (A) Formation of chasing pairs within the consumption process between consumers and resource. (B) Formation of chasing triplet involving two consumers and a resource. (C, E) Consumer species cannot coexist at a steady state in the scenario involving only chasing pairs. (D, F) Coexistence of consumers in the scenario involving both chasing pairs and chasing triplets. The arrows in (E, F) indicate the directions of time evolution. Figure redrawn from the study by Wang and Liu [12]. (C–F) were produced using the same equations as in the reference [12], but with slightly different parameters. CEP, competitive exclusion principle.

The model for the simple case of two consumer species competing for one resource species (*S*_*C*_ = 2 and *S*_*R*_ = 1) is shown in Figure 2A [12], where the population structure of the consumers and resources is explicitly modeled: $C_{i}^{\left(F\right)}$ and *R* ^(F)^ represent the consumers and resources that are freely wandering (represented by the superscript ‘(F)’). When a consumer and a resource individual get close in space, the consumer chases the resource and they form a chasing pair $x_{i}=C_{i}^{\left(P\right)}\vee R^{\left(P\right)}$ (the superscript ‘(P)’ represents pair). Then, the population abundances of consumer species *C*_*i*_ and resource species *R* are $C_{i}=C_{i}^{\left(F\right)}+x_{i}$ and $R=R^{\left(F\right)}+\sum_{i=1}^{2}x_{i}$, respectively. The population dynamics is modeled as follows [12]:

(4) $$\left\{\begin{array}{l} {\dot{x}}_{i}=a_{i}C_{i}^{\left(F\right)}R^{\left(F\right)}-\left(d_{i}+k_{i}\right)x_{i},\\ {\dot{C}}_{i}=w_{i}k_{i}x_{i}-D_{i}C_{i},\\ \dot{R}=g_{1}\left(R,C_{1},C_{2},x_{1},x_{2}\right),i=1,2, \end{array}\right.$$

where *a*_*i*_, *d*_*i*_, *k*_*i*_, *w*_*i*_ and *D*_*i*_ are model parameters and *g*_1_ represents a unspecified function. At a steady state, if both consumer species coexist, it requires that *f*_*i*_ (*R* ^(F)^)/*D*_*i*_ = 1 (*i* = 1, 2), where $f_{i}\left(R^{\left(F\right)}\right)=\frac{w_{i}\cdot k_{i}\cdot R^{\left(F\right)}}{R^{\left(F\right)}+K_{i}}$ and $K_{i}=\frac{d_{i}+k_{i}}{a_{i}}$ (*i* = 1, 2). This demands that two curves *y* = *f*_1_ (*R* ^(F)^)/*D*_1_ and *y* = *f*_2_ (*R* ^(F)^)/*D*_2_ intersect the line *y* = 1 at the same point, rendering coexistence typically impossible (see Figure 2C,E) [12].

## 4 MECHANISMS LIBERATING THE CONSTRAINT OF CEP THROUGH CONTEXTUAL DIFFERENCES

Below, we introduce mechanisms promoting biodiversity by liberating the constraint of CEP. First, we examine those achieving this through contextual differences.

### 4.1 Temporal variations in the environment

**FIGURE 3:**
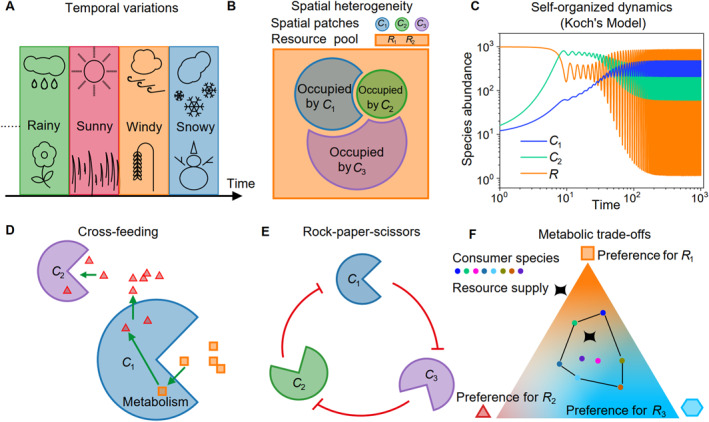
Mechanisms overcoming the constraint set by CEP through contextual differences. (A) Temporal variations in the environments [22, 23]. (B) Spatial heterogeneity or patchiness [24]. (C) Self‐organized dynamics such as oscillating coexistence [25]. (D) An illustration of cross‐feeding among microbial species [26]. (E) An illustration of the rock–paper–scissors relationship among consumer species [27]. (F) Metabolic trade‐offs in microbial communities. With different resource preferences, multiple consumers may coexist in stochastic simulation studies when the external resource supply lies within the convex hull of the consumers, a closed region delineated by black lines [28]. CEP, competitive exclusion principle.

In 1961, in a famous work on the “Paradox of Plankton,” Hutchinson proposed that temporal variation could be a crucial factor for bypassing the constraint of CEP in the wild [22]. Indeed, temporal variations are widespread in nature, such as day–night cycles, seasonal shifts, and unpredictable weather variations such as alternations among precipitation, sunshine, wind, and snowfall (refer to Figure 3A). Advantageous species could vary with changing environmental factors, and thus, if competition does not lead to species extinction before environmental changes occur, then equilibrium is never reached, allowing species to bypass the CEP due to this factor [22].

In the subsequent studies, Levins proposed that consumer populations not only exploit resources but also create new ecological niches amidst temporal variability and nonlinear dynamics [23]. This process may hinder ecosystems from attaining stable equilibrium. In 2006, Adler et al. conducted a meticulous examination of 3 decades of demographic data from a Kansas prairie, unveiling how interannual climate variability facilitates the coexistence of three dominant grass species [29]. This climate variability enhances growth rates at low densities, thereby preventing competitive exclusion.

### 4.2 Spatial heterogeneity

Spatial heterogeneity is a common feature of ecological environments, presenting as a mosaic of diverse subenvironments inhabited by different species (see Figure 3B) rather than adhering to a uniformly mixed, homogeneous condition. In 1974, Levin proposed that spatial heterogeneity contributes to increased species diversity [24]. This concept can be illustrated using the example depicted in Figure 3B, where three consumer species (*C*_1_, *C*_2_, and *C*_3_) compete for two resource species (*R*_1_ and *R*_2_) within an area comprising three distinct patches. If each patch is occupied by a different consumer species, then, although each patch is subject to the constraint of the CEP, when considered as a whole, it is evident that there are more consumer species than resource species (Figure 3B). Furthermore, Levin elucidated that heterogeneity can emerge in an initially homogeneous environment due to random initial events, such as colonization patterns, with their effects magnified by species interactions.

The influence of spatial heterogeneity is widely documented in studies of natural ecosystems. Within plant communities, the spatial arrangement of various species often exhibits gaps and patches [30, 31]. In aquatic ecosystems, variations in factors such as light exposure and temperature can lead to chemical stratification across different depths [32]. For plankton communities, sampling studies in the 1970s at Castle Lake, California, revealed significant patchiness among various phytoplankton species [33]. In 2015, expanded research conducted during the Tara Oceans expedition collected ocean samples worldwide, revealing diversified planktonic communities and significant vertical stratification mainly driven by temperature differences in depth [4, 34]. Recently, spatial heterogeneity incorporating ocean currents has been included in modeling studies [35, 36].

### 4.3 Self‐organized dynamics

For well‐mixed systems in a stable environment, self‐organized dynamics may naturally arise from species interactions, leading to species coexistence in an oscillatory or chaotic manner. This disrupts the attainment of an equilibrium state and bypasses the constraint of CEP (see Figure 3C).

Specifically, in 1974, Koch proposed a model for the case involving two species of consumers and one species of biotic resources (*S*_*C*_ = 2 and *S*_*R*_ = 1), and demonstrated that in deterministic simulation studies, both consumer species could coexist through oscillations (Figure 3C), naturally breaking the constraint of CEP by bypassing a steady state [25]. In 1999, Huisman and Weissing [37] conducted modeling studies for systems involving multiple resource species and demonstrated the enduring coexistence of many consumer species through oscillations and chaos in the population dynamics with deterministic simulation studies.

In experimental systems, evidence was reported on the role of self‐organized dynamics in promoting biodiversity. For instance, in 2005, Becks et al. reported the coexistence of both oscillating and chaotic behaviors of microbial species in chemostat systems [38]. In 2008, Benincà et al. reported experimental observations of chaos in the analysis of a 6‐year long‐term coexistence of a plankton community from the Baltic Sea and suggested that chaotic behavior could be a plausible explanation for the paradox of plankton [39].

### 4.4 Cross‐feeding

Cross‐feeding is a prevalent phenomenon in microbial ecosystems, where microbial species excrete metabolites that serve as nutrients into the culture medium or their surrounding environment. This process leads to the production of additional resources beyond those initially provided (see Figure 3D). For example, in the illustration shown in Figure 3D, there is only one type of externally supplied resource, yet two consumer species may coexist at a steady state because species *C*_1_ produces another type of resource by excreting metabolites. Consequently, if we only count the externally supplied types of resources, the microbial ecosystem appears to violate the CEP. However, when the resources secreted by microbes are included in the total types of resources, this mechanism actually operates within the constraints of the CEP.

Cross‐feeding stands out as one of the most effective ways to promote biodiversity in microbial ecosystems. Goyal and Maslov employed model simulation studies to illustrate that microbial ecosystems exhibit high diversity, stability, and partial reproducibility even with only one type of resource owing to cross‐feeding [40]. In 2018, Goldford et al. reported experimental findings highlighting the widespread occurrence of nonspecific cross‐feeding in microbial communities. They observed the stable coexistence of multiple microbial species with the external supply of just a single type of carbon source [26].

More recently, Lopez and Wingreen introduced a theory known as the noisy metabolism‐averaging theory, aimed at elucidating the emergence of inter‐species cross‐feeding. Their theory is grounded in the premise that bacteria, owing to their small size, are vulnerable to the noisy regulation of metabolism, thereby constraining their growth rate. To counteract this limitation, closely related bacteria can engage in the sharing of metabolites to “average out” noise and bolster their collective growth [41].

### 4.5 Complex interspecific interactions

In the wild, species may coexist through complex interactions such as rock–paper–scissors relations and higher‐order interactions. The rock–paper–scissors relation is observed in ecosystems where three species, *C*_1_, *C*_2_, and *C*_3_, follow the rule where rock dominates scissors, scissors prevail over paper, and paper trumps rock (see Figure 3E). This intransitive competition relationship ensures that species emerge victorious in at least one pairwise competition, thereby preventing the dominance of a single all‐winning species and promoting coexistence [42].

The rock–paper–scissors relation is commonly observed in bacterial communities because of the additional effects of antibiotic excretions and antibiotic resistance [27, 43, 44], which may result in either stable or self‐organized oscillatory coexistence in microbial communities. This relation has also been reported in macroscopic natural ecosystems, such as the cryptic coral reef system [45] and lizard communities [46]. However, temporal environmental change and demographic factors may also play a role in affecting species competitiveness in the wild, which may thus shape the rock–paper–scissors relation.

Higher‐order interaction implies that the presence of a third species modifies the original interaction between two other species. For example, if a toxin produced by species *C*_1_ inhibits species *C*_2_, while species *C*_3_ is capable of degrading this toxin, then it exerts a higher‐order interaction. In 2015, Kelsic et al. studied the integration of high‐order interaction with the rock–paper–scissors relation in microbial communities and revealed a broad spectrum of coexistence patterns through modeling studies ranging from stable coexistence, oscillations to chaos, when multiple antibiotics are involved [47]. In 2016, Bairey et al. found that higher‐order interaction can facilitate ecosystems incorporating a high level of species diversity to stably coexist [48]. This is validated by Grilli et al.’s theoretical studies [49] in which they revealed the stabilizing role of higher‐order interaction, turning oscillatory and chaotic coexistence into stable coexistence, thereby increasing the vulnerability of ecosystems.

### 4.6 Metabolic trade‐offs

Metabolic trade‐offs refer to the constraints faced by consumer species due to a trade‐off in resource allocation for feeding on different resource species [28]. In microbial communities, protein allocation is a notable constraint leading to metabolic trade‐offs. In 2017, Posfai et al. introduced a pioneering model incorporating this mechanism, elucidating that with the introduction of stochasticity, multiple microbial species can coexist with three types of resources when the external supply of resources falls within a certain range of resource preference region set by the consumer species (see Figure 3F) [28].

In practice, the deterministic version of Posfai et al.’s model [28] satisfies the functional form considered in MacArthur and Levin’s proof of the CEP [7]. Meanwhile, stochasticity is prone to jeopardize the coexistence of species in ecological models [50]. However, for the mechanism of metabolic trade‐offs, stochasticity plays a role similar to that of stochastic resonance [51], facilitating species coexistence through the integration of metabolic trade‐offs, thereby promoting biodiversity in microbial ecosystems.

### 4.7 Food web

In nature, a food web represents the complex interaction network among various organisms, playing crucial roles in shaping the stability and diversity of ecological communities [52, 53, 54]. In a food web, species at an intermediate trophic level feed on food at a lower trophic level; yet they can be preyed upon by consumers at a higher trophic level. This allows for the number of species at an upper trophic level to exceed the number of species at the adjacent lower trophic level, apparently breaking the CEP. The most notable example is the mechanism of “Kill the winner” [55], where viruses lyse the most advantageous bacteria, leading to a competitive balance between different bacterial species and thus facilitating species coexistence.

Similar examples have also been reported in many other systems [56, 57]. Ceulemans et al. showed that in a three‐trophic‐level system, the presence of a top‐level consumer can disrupt CEP between intermediate and basal level species, highlighting the significant role played by top‐level consumers in maintaining ecological diversity and stability within food webs [57].

### 4.8 Neutral theory

The neutral theory in ecology, commonly referred to as Hubbell’s “The Unified Neutral Theory of Biodiversity and Biogeography,” is a highly impactful theory in explaining biodiversity. Most notably, it broadly illustrates the rank abundance curves across a wide variety of ecological communities. This theory is based on the symmetry assumption or “Neutrality,” which posits that all species at a given trophic level in a food web share exactly the same rates of birth, death, migration, and speciation on a per‐capita basis [58, 59]. Under the neutral theory, a plethora of consumer species may coexist at a steady state with a few types of resources.

Despite the success of the application of neutral theory for illustrating ecological data across communities, the appropriateness of the “Neutrality” assumption has drawn considerable controversy [60, 61, 62]. All its predictions and conclusions heavily rely on the identical property assumption across organisms and individuals [59]; even slight differences among individuals would invalidate all its predictions [62]. From a probability perspective, the requirement of neutral theory corresponds to a zero‐measure of parameter space, which is the main idea MacArthur and Levin used in the classical proof of the CEP [7].

### 4.9 Coexistence theory

Building on the GLV model framework, Chesson developed the modern coexistence theory to explain species diversity in nature [63, 64, 65]. Despite its similarity to the GLV model in equation form, a major difference introduced by the coexistence theory is the inclusion of the temporal influence of the environment on the per capita growth rate [64, 65].

The coexistence theory has clarified two key mechanisms for promoting species coexistence: equalizing, which minimizes average fitness differences between consumer species and stabilizing, which promotes coexistence by reducing niche overlap, that is, “encouraging” intraspecific competition rather than interspecific competition [64, 66]. For instance, resource partitioning, where consumer species each have an advantage for specific resources, is a typical example of stabilizing, which promotes species coexistence yet still operates subject to the constraint of the CEP. The storage effect [67] is another type of stabilizing, where consumer species are impacted differently by environmental variation across space or time. It incorporates mechanisms such as temporal variations or spatial heterogeneity, facilitating species coexistence and breaking the CEP [64, 65].

## 5 MECHANISMS BREAKING CEP IN ITS ORIGINAL CONTEXT

Below, we review mechanisms for overcoming CEP in its original context within the framework of well‐mixed systems operating at a steady state.

### 5.1 Chasing triplets

Pack hunting is a phenomenon commonly observed across different organisms [68, 69, 70, 71, 72, 73, 74, 75, 76] wherein multiple consumer individuals pursue a resource individual simultaneously. Building on these observations, Wang and Liu proposed a model that extended the consideration of chasing pairs to chasing triplets [12], where a consumer can join an existing chasing pair $x_{i}=C_{i}^{\left(P\right)}\vee R^{\left(P\right)}$ to form a chasing triplet $y_{i}=C_{i}^{\left(T\right)}\vee R^{\left(T\right)}\vee C_{i}^{\left(T\right)}$ (see Figure 2B, the superscript ‘(T)’ represents triplet). For a system containing two consumer species and one type of abiotic resource (*S*_*C*_ = 2 and *S*_*R*_ = 1), the population abundances of consumers and resources are $C_{i}=C_{i}^{\left(F\right)}+x_{i}+2y_{i}$ and $R=R^{\left(F\right)}+\sum_{i=1}^{2}\left(x_{i}+y_{i}\right)$ (*i* = 1, 2), and the population dynamics is modeled as follows [12]:

(5) $$\left\{\begin{array}{l} {\dot{x}}_{i}=a_{i}C_{i}^{\left(F\right)}R^{\left(F\right)}-\left(d_{i}+k_{i}\right)x_{i}-b_{i}x_{i}C_{i}^{\left(F\right)}+e_{i}y_{i},\\ {\dot{y}}_{i}=b_{i}x_{i}C_{i}^{\left(F\right)}-\left(h_{i}+e_{i}+l_{i}\right)y_{i}\\ {\dot{C}}_{i}=w_{i}\left(k_{i}x_{i}+h_{i}y_{i}\right)-D_{i}C_{i},\\ \dot{R}=R_{a}\left(1-R/K_{a}\right)-\sum_{i=1}^{2}k_{i}x_{i}-\sum_{i=1}^{2}h_{i}y_{i},i=1,2. \end{array}\right.$$

where *a*_*i*_, *b*_*i*_, *d*_*i*_, *e*_*i*_, *h*_*i*_, *k*_*i*_, *l*_*i*_, *w*_*i*_, *D*_*i*_, *R*_*a*_, and *K*_*a*_ are model parameters. At a steady state, the zero‐growth isoclines of the three species (i.e., ${\dot{C}}_{1}=0$, ${\dot{C}}_{2}=0$, and $\dot{R}=0$) correspond to nonparallel surfaces in the (*C*_1_, *C*_2_, and *R*) coordinates, which shares a common point (see Figure 5E). Such a fixed point can be stable, and hence the mechanism of chasing triplets enables two consumer species to coexist with one type of resources at steady state (see Figure 2D,F), thereby breaking CEP in its original context [12].

### 5.2 Intraspecific predator interference

Predator interference refers to the pairwise encounters between consumer individuals, encompassing interactions ranging from subtle staring contests to overt physical confrontations. In 1975, both Beddington [78] and DeAngelis et al. [79] independently introduced identical phenomenological models to elucidate the influence of predator interference on the functional response of consumer species. These models were later collectively referred to as the Beddington–DeAngelis (B‐D) model by subsequent studies. The functional response of the B‐D model is represented as follows:

(6) $$F\left(R,C_{i}\right)=\frac{aR}{1+at_{h}R+a^{\prime }t_{w}C_{i}},$$

where *a*, *a*′, *t*_*h*,_ and *t*_*w*_ are model parameters. Note that the functional response described above Equation (6) includes consumer dependency and thus does not conform to the functional form outlined in MacArthur and Levins’ classical proof [7]. Consequently, the B‐D model has been used in several studies to liberate the constraint of CEP [80, 81]. However, it is worth noting that the functional response of the B‐D model can be derived from scenarios involving only chasing pairs without predator interference [12, 82]. This casts doubt on the appropriateness of employing the B‐D model to break the CEP, as scenarios involving only chasing pairs are subject to the constraints of CEP [12].

**FIGURE 4:**
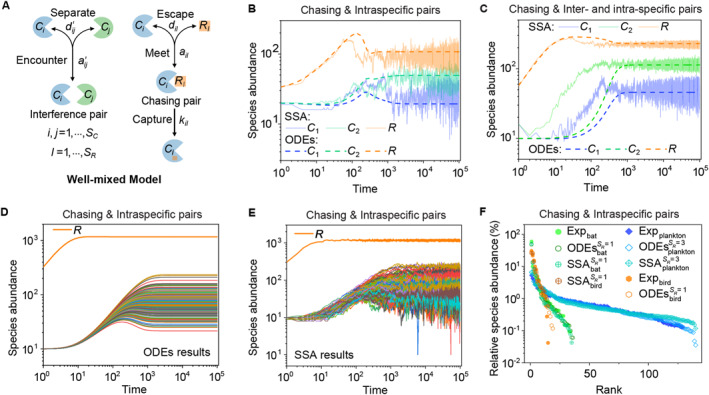
A mechanistic model of intraspecific predator interference breaks the CEP and promotes biodiversity [77]. (A) Kang et al.’s mechanistic model of pairwise encounters. (B–C) Intraspecific predator interference facilitates the break of CEP. (D–E) Intraspecific interference enables multiple consumer species to coexist with one type of resource species irrespective of stochasticity. (F) Kang et al.’s model of intraspecific predator interference quantitatively illustrates the species’ rank‐abundance curves across diverse ecological communities. Figure redrawn from the study by Kang et al. [77]. (B–E) were produced using the same equations as in the reference [77], but with slightly different parameters. CEP, competitive exclusion principle.

Most recently, Kang et al. developed a mechanistic model of predator interference to address this issue [77], wherein they applied mean field theory to model the pairwise encounters between consumer and resource individuals (see Figure 4A). For a system containing *S*_*C*_ consumer species and *S*_*R*_ types of abiotic resources, considering a scenario involving chasing pairs and both intra‐ and interspecific predator interference, the population dynamics are modeled as follows:

(7) $$\left\{\begin{array}{l} {\dot{x}}_{il}=a_{il}C_{i}^{\left(F\right)}R_{l}^{\left(F\right)}-\left(d_{il}+k_{il}\right)x_{il},\\ {\dot{z}}_{ii}=a_{ii}^{\prime }{\left[C_{i}^{\left(F\right)}\right]}^{2}-d_{ii}^{\prime }z_{ii},\\ {\dot{z}}_{ij}=a_{ij}^{\prime }C_{i}^{\left(F\right)}C_{j}^{\left(F\right)}-d_{ij}^{\prime }z_{ij},i\neq j,\\ {\dot{C}}_{i}=\sum_{l=1}^{S_{R}}w_{il}k_{il}x_{il}-D_{i}C_{i},\\ {\dot{R}}_{l}=R_{a}^{\left(l\right)}\left(1-R/K_{a}^{\left(l\right)}\right)-\sum_{i=1}^{S_{C}}k_{il}x_{il},i=1,\text{⋯}S_{C},l=1,\text{⋯}S_{R}, \end{array}\right.$$

where $x_{il}=C_{i}^{\left(P\right)}\vee R_{l}^{\left(P\right)}$ represents a chasing pairs, and $z_{ij}=C_{i}^{\left(P\right)}\vee C_{j}^{\left(P\right)}$ stands for a predator interference pair (with *i = j* for intraspecific and *i* ≠ *j* for interspecific), $C_{i}=C_{i}^{\left(F\right)}+\sum_{l}x_{il}+2z_{ii}+\sum_{j\neq i}z_{ij}$ and $R_{l}=R_{l}^{\left(F\right)}+\sum_{i}x_{il}$, while *a_il_*, *d*_*il*_, *k*_*il*_, $a_{ij}^{\prime }$, $d_{ij}^{\prime }$, *w*_*il*_, *D*_*i*_, $R_{a}^{\left(l\right)}$, and $K_{a}^{\left(l\right)}$ are model parameters. Using this model, Kang et al. demonstrated that with the mechanism of intraspecific predator interference, two or more, even hundreds of consumer species can coexist at steady state when there is only one type of abiotic resource (see Figure 4B–E). Furthermore, such coexistence states are robust to stochasticity (see Figure 4B,C,E), and this model [77] can even quantitatively illustrate the rank abundance curves observed in diversified ecological communities (see Figure 4F), including communities of plankton [83, 84], birds [85, 86, 87], bats [88], and many other organisms in the wild.

## 6 A NECESSARY CONDITION FOR MECHANISMS BREAKING CEP IN ITS ORIGINAL CONTEXT

Intuitively, there is a quick test to check if a mechanism can break CEP in its original context. Consider the simplest case involving two consumer species *C*_1_ and *C*_2_, and one resource species *R* (*S*_*C*_ = 2 and *S*_*R*_ = 1). The zero‐growth isoclines of the three species, that is, ${\dot{C}}_{1}=0$, ${\dot{C}}_{2}=0$, and $\dot{R}=0$, correspond to three planes or surfaces in the (*C*_1_, *C*_2_, and *R*) coordinates. A necessary condition for a mechanism to break CEP in its original context is that all these planes or surfaces are nonparallel to each other.

**FIGURE 5:**
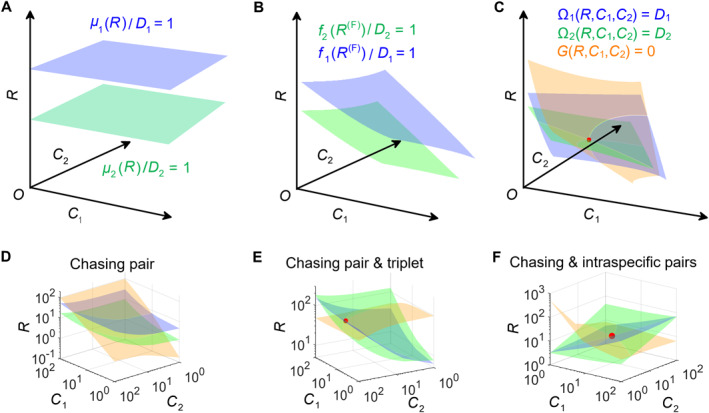
Intuitive understanding of mechanisms breaking CEP in its original context. Here, the blue surface, green surface, and orange surface represent the zero‐growth isoclines of species *R*, *C*_1,_ and *C*_2_, respectively. (A) In MacArthur and Levins’ classical proof [7], the isoclines of species *C*_1_ and *C*_2_ correspond to two parallel planes. (B, D) In chasing the pair model [12], the isoclines of species *C*_1_ and *C*_2_ correspond to two parallel surfaces. (C) A necessary condition for mechanisms breaking CEP at steady state is that the three surfaces have a common point. (E, F) Examples of the chasing triplet model [12] or mechanistic model of intraspecific predator interference [77]; the fixed points shown in red are stable. Figure redrawn from the study by Wang and Liu [12] and Kang et al. [77]. (D–F) were produced using the same equations as in the references [12, 77] but with slightly different parameters. CEP, competitive exclusion principle.

For instance, in MacArthur and Levins’ classical proof of the CEP [7], if all species coexist, the zero‐growth isoclines of species *C*_1_ and *C*_2_ correspond to two parallel planes governed by *μ*_*i*_(*R*)/*D*_*i*_ = 1 (*i* = 1, 2, see Equation (1) and Figure 5A), which do not share a common point. Thus, a system described by Equation (1) is subject to the constraint of CEP. In a scenario involving only chasing pairs, the zero‐growth isoclines of species *C*_1_ and *C*_2_ correspond to two parallel surfaces governed by *f*_*i*_ (*R* ^(F)^)/*D*_*i*_ = 1 (*i* = 1, 2, see Equation (4) and Figure 5B,D). Therefore, this scenario is also under the constraint of CEP [12]. For scenarios involving chasing triplets or intraspecific predator interference, the zero‐growth isoclines of the three species correspond to three nonparallel surfaces and thus share a common point (see Equations (5) and (7), and Figure 5C,E,F). For these two mechanisms, the fixed points are stable, and thus both mechanisms [12, 77] can break CEP at steady state in the original context.

## 7 CONCLUSION

The challenge posed by the CEP to biodiversity, as highlighted by the paradox of the plankton, has attracted intense research interest over the past several decades. In this review, we mainly introduce mechanisms that promote biodiversity by alleviating the constraints imposed by the CEP. Generally, these mechanisms can be classified into two different categories based on whether they adhere to the original context of the CEP.

The first category of these mechanisms breaks the constraint set by the CEP through circumventing its limitations via contextual differences. Among these, the most notable are mechanisms that assert ecosystems in nature never reach a steady state due to temporal variations [22, 23, 89], spatial heterogeneity [24, 33], or self‐organized dynamics such as oscillating and chaotic coexistence [25, 37, 39]. The first category also includes mechanisms such as cross‐feeding, which operates within the constraint of the CEP if all secreted resources are counted; yet it plays an important role in maintaining species diversity, especially in microbial ecosystems. Metabolic trade‐off [28] is also a mechanism that may contribute to biodiversity in microbial ecosystems, based on its validity in promoting biodiversity when stochasticity is introduced. The neutral theory [58] is extremely successful in explaining species distribution data on rank abundance curves across ecological communities, despite its special “Neutrality” requirement for the parameters.

The second category of mechanisms breaks the CEP in its original context involving well‐mixed systems and steady states. This includes mechanisms such as chasing triplets [12] and intraspecific predator interference [77, 78, 79]. For this type of category, special attention needs to be paid since approximations in the functional response may lead to errors in breaking the CEP. As mentioned earlier, the functional response of the B‐D model [78, 79] involving intraspecific predator interference could also be derived from scenarios involving only chasing pairs [12, 82]. Since a scenario involving only chasing pairs is under the constraint of the CEP [12], it would inevitably cast doubt on the appropriateness of applying the B‐D model [78, 79] to break the CEP. Fortunately, Kang et al.’s study resolved this issue and proved that intraspecific predator interference could truly break the CEP and potentially resolve the paradox of the plankton based on rigorous examination [77]. Ultimately, a necessary condition for a mechanism to break the CEP while adhering to the original context requires that in the case of two consumer species competing for one resource species, the zero‐growth isoclines of the three species should correspond to three nonparallel surfaces which share a common point (see Figure 5C,E,F), and once the fixed point is stable, there would be a rigorous breaking of CEP in its original context.

Beyond mechanisms promoting biodiversity by alleviating the constraints set by the CEP, there are also many other mechanisms promoting species diversity through stabilizing species coexistence, such as higher‐order interactions and rock–paper–scissors relations, where secreted metabolites or toxins are commonly involved in species interactions. For promoting biodiversity in natural ecosystems, many of the mechanisms mentioned above are potentially contri‐buting, sometimes overlapping. It remains a challenge to identify the leading factors that promote biodiversity for specific real ecosystems.

## AUTHOR CONTRIBUTIONS

**Ju Kang**: Data curation; formal analysis; investigation; software; validation; visualization; writing—original draft. **Yiyuan Niu:** Data curation; formal analysis; investigation; software; validation; visualization; writing—original draft. **Xin Wang**: Conceptualization; formal analysis; funding acquisition; investigation; project administration; supervision; validation; writing—original draft; writing—review & editing.

## CONFLICT OF INTEREST STATEMENT

The authors declare that they have no conflicts of interest.

## ETHICS STATEMENT

This review article does not involve any research related to human or animal subjects.

## Data Availability

The data that support the findings of this study are available from the corresponding author upon reasonable request.
